# Supplementary material for: TinyAct: A framework for real-time action recognition in the cloud through distillation learning
Source: PLoS One. 2026 Apr 20;21(4):e0347245. doi: 10.1371/journal.pone.0347245 (PMC13095028; doi:10.1371/journal.pone.0347245)
Supplement: Attachment — Submitted filename: TinyAct-Response-Letter-27-Feb-2026.pdf [file pone.0347245.s001.pdf]

Response to Reviewers and Editorial Requirements

Part A: Response to Editorial Requirements

1. Please ensure that your manuscript meets PLOS ONE's style requirements, including those for file naming. The PLOS ONE style templates can be found at
https://journals.plos.org/plosone/s/file?id=wjVg/PLOSOne_formatting_sample_main_body.pdf and
https://journals.plos.org/plosone/s/file?id=ba62/PLOSOne_formatting_sample_title_authors_affiliations.pdf

2. Please note that PLOS One has specific guidelines on code sharing for submissions in which author-generated code underpins the findings in the manuscript. In these cases, we expect all author-generated code to be made available without restrictions upon publication of the work. Please review our guidelines at https://journals.plos.org/plosone/s/materials-and-software-sharing#loc-sharing-code and ensure that your code is shared in a way that follows best practice and facilitates reproducibility and reuse.

3. Funding Statement Clarification and 4. Acknowledgments Section Revision
Thank you for requesting clarification on funding disclosure. We have addressed both requirements together as they relate to the same issue:

<u>Response:</u>

Thank you for requesting clarification on funding disclosure. We have addressed both requirements as follows:

<u>1. COMPLETE FUNDING STATEMENT (Requirements #3 & #4):</u>

We confirm that both funding sources provided financial support only and had no involvement in the research process or publication decisions.

<u>ADDED FUNDING STATEMENT:</u>

"This study was financially supported by the Fundamental Fund of Khon Kaen University (fiscal year 2024, project number 200553) and the National Science, Research and Innovation Fund (NSRF), Thailand. The funders had no role in study design, data collection and analysis, decision to publish, or preparation of the manuscript."

<u>2. ACKNOWLEDGMENTS SECTION REVISION:</u>

We have removed all funding-related text from the Acknowledgments section (page 15, lines 535-538) as per PLOS ONE policy.

5. We note that Figure 1 includes an image of a [patient / participant / in the study].
As per the PLOS ONE policy (http://journals.plos.org/plosone/s/submission-guidelines#loc-human-subjects-research) on papers that include identifying, or potentially identifying, information, the individual(s) or parent(s)/guardian(s) must be informed of the terms of the PLOS open-access (CC-BY) license and provide specific permission for publication of these details under the terms of this license. Please download the Consent Form for Publication in a PLOS Journal (http://journals.plos.org/plosone/s/file?id=8ce6/plos-consent-form-english.pdf). The signed consent form should not be submitted with the manuscript, but should be securely filed in the individual's case notes. Please amend the methods section and ethics statement of the manuscript to explicitly state that the patient/participant has provided consent for publication: “The individual in this manuscript has given written informed consent (as outlined in PLOS consent form) to publish these case details”.

If you are unable to obtain consent from the subject of the photograph, you will need to remove the figure and any other textual identifying information or case descriptions for this individual.

Response:

Thank you for raising this important concern regarding ethical considerations for images in Figure 1.

The images shown in Figure 1 are NOT photographs of participants in our study. They are sample frames from the publicly available Kinetics-400 dataset [Kay et al., 2017], a standard benchmark widely used and cited in computer vision research. No human subjects were enrolled, observed, or recorded as part of this research. All experiments were conducted exclusively using the pre-existing Kinetics-400 dataset.

Despite the public availability of these images, we have taken the following precautionary measures in the revised manuscript:

1. FACIAL OBSCURING: All facial regions visible in Figure 1 have been blurred to prevent potential identification of individuals appearing in the dataset samples.

2. FIGURE CAPTION UPDATED: The caption of Figure 1 now includes a note indicating that the video frames are illustrative samples from the Kinetics-400 dataset with obscured facial regions.

Part B: Response to Reviewer #1

	Comment	Response
1	Comment 1: The paper presents a compelling solution to the critical challenge of deploying real-time action recognition on resource-constrained edge devices. The combination of edge computing with cloud-based processing through knowledge distillation represents a practical approach to balance computational efficiency with accuracy requirements. Please take assistance from the study titled ***"efficient deepfake detection via layer-frozen assisted dual attention network for consumer imaging devices"*** for optimizing lightweight architectures on consumer-grade edge devices.	We thank the reviewer for recognizing the practical value of our edge-cloud approach and for this excellent suggestion. We have incorporated insights from Usman et al.'s work on efficient architectures for consumer devices, which provides valuable perspectives on balancing efficiency and accuracy in resource-constrained environments.<br>CHANGES IN MANUSCRIPT:<br>1. References (Page 22, Ref [16])<br>Added: Usman MT, Khan H, Singh SK, Lee MY, Koo J. Efficient deepfake detection via layer-frozen assisted dual attention network for consumer imaging devices. IEEE Transactions on Consumer Electronics. 2024;71(1):281–291.<br>2. Related Work (Page 3, Lines 85–97)<br>Added a new paragraph in the "Lightweight Neural Network Architectures for Edge Devices" subsection discussing Usman et al.'s layer-frozen dual attention approach using EfficientNetV2 with CBAM, and contrasting their spatial artifact detection focus with TinyAct's temporal modeling for action recognition.<br>3. Methodology (Page 10, Lines 343–364)<br>Added new subsection "Layer-Freezing Considerations for Temporal Feature Learning" describing two training configurations inspired by [16]: (1) frozen encoder with only MLP fine-tuned, and (2) joint end-to-end optimization. Results show frozen encoder achieved only 5.82% vs 15.11% for joint optimization, revealing that action recognition requires encoder-classifier co-adaptation unlike static image tasks.<br>4. Future Work (Page 20, Lines 766–774)<br>Added future directions including lightweight attention mechanisms and progressive unfreezing strategies to narrow the KD performance gap and strengthen the framework.
2	Comment 2: The AIoT architecture design is well-structured, clearly separating edge device responsibilities (data collection, preprocessing, feature extraction) from cloud processing (action classification, storage, visualization). This separation enables effective resource utilization while maintaining real-time processing capabilities. Please take assistance from the study titled	We thank the reviewer for recognizing the effectiveness of our AIoT architecture design and for this valuable suggestion regarding efficient attention mechanisms. We have incorporated insights from recent work on lightweight multi-scale attention, which provides complementary perspectives on temporal feature processing for edge deployment.<br>CHANGES IN MANUSCRIPT:<br>1. ADDED NEW REFERENCE (Page 22, Ref [18])

	"lightweight transformer-driven multi-scale trapezoidal attention network for saliency detection" for implementing efficient attention mechanisms in the edge processing pipeline.	Added: Usman MT, Khan H, Rida I, Koo J. Lightweight transformer-driven multi-scale trapezoidal attention network for saliency detection. Engineering Applications of Artificial Intelligence. 2025;155:110917.<br>2. EXPANDED RELATED WORK SECTION — Page 4, Section "Lightweight Neural Network Architectures for Edge Devices", Lines 106–115<br>Added a paragraph discussing how multi-scale trapezoidal attention [18] demonstrates efficient feature extraction through hierarchical attention that progressively narrows scope across layers, reducing complexity compared to full self-attention. We explain that while such mechanisms could identify motion-salient frames for action recognition, TinyAct currently prioritizes inference speed via 3D autoencoder compression, reserving attention mechanisms as a future enhancement.<br>3. ENHANCED FUTURE WORK SECTION — Page 20, Conclusion section, Future Work paragraph, Lines 766–774<br>Added "lightweight attention mechanisms" as a future direction for narrowing the KD performance gap through intermediate feature distillation and progressive unfreezing strategies to strengthen the framework.
3	<u>Comment 3:</u> The knowledge distillation approach using ILA (Intermediate Layer Alignment) as a teacher model to transfer temporal knowledge to the compact student architecture is technically sound. However, the paper lacks detailed explanation of how the distillation process handles the temporal complexity inherent in action recognition tasks. Please take assistance from the study titled ***"optimal features driven hybrid attention network for effective video summarization"*** for better temporal feature extraction and knowledge transfer strategies.	We thank the reviewer for this insightful comment. We acknowledge that the original manuscript did not sufficiently address how our knowledge distillation process handles temporal complexity in action recognition. Following the reviewer's valuable suggestion, we have carefully studied Khan et al.'s work on hybrid attention-based video summarization and incorporated relevant insights into our revised manuscript.<br><u>CHANGES IN MANUSCRIPT:</u><br>1. ADDED NEW REFERENCE (Page 23, Ref [30])<br>Added: Khan H, Khan SU, Ullah W, Baik SW. Optimal features driven hybrid attention network for effective video summarization. Engineering Applications of Artificial Intelligence. 2025;158:111211.<br>2. EXPANDED KNOWLEDGE DISTILLATION SECTION (Methodology)<br>We have substantially expanded the Knowledge Distillation section with two new subsections totaling approximately 2.5 pages:

		New Subsection: "Handling Temporal Complexity in Knowledge Distillation"<br>Location: Page 13–14, Lines 476–533<br>— Covers temporal feature alignment, progressive temporal learning, hybrid feature refinement for temporal patterns, and reconstruction as temporal regularization. Cites [30] for hybrid attention mechanisms.<br>New Subsection: "Temporal Knowledge Transfer Strategy"<br>Location: Page 15–16, Lines 568–594<br>— Describes the staged training approach: warm-up phase (20 epochs, reconstruction + ground-truth only), full distillation phase (T=4), and joint optimization rationale explaining why non-pretrained students (15.11%) outperform frozen encoders (5.82%).<br>3. ENHANCED FUTURE WORK SECTION — Page 20, Lines 766–774<br>Added future directions on intermediate feature distillation, lightweight attention mechanisms, and attention-based transfer strategies to narrow the KD performance gap.
4	Comment 4: The experimental validation on Kinetics-400 dataset demonstrates competitive performance (57.00% accuracy) compared to larger models, but the evaluation appears limited to a single dataset. ***Testing on additional action recognition benchmarks (UCF-101, HMDB-51) is necessary to establish generalizability.*** Please take assistance from the study titled “bilateral feature fusion with hexagonal attention for robust saliency detection under uncertain environments” for handling diverse environmental conditions and dataset variations.	We thank the reviewer for this valuable suggestion regarding multi-dataset evaluation. We acknowledge that testing on additional benchmarks such as UCF-101 and HMDB-51 would further strengthen the generalizability assessment of TinyAct.<br>In the current study, we selected Kinetics-400 as our primary benchmark because it offers significantly greater scale and diversity — comprising 400 action categories with approximately 246,000 video clips — compared to UCF-101 (101 classes) and HMDB-51 (51 classes). This makes Kinetics-400 one of the most challenging and comprehensive benchmarks for evaluating action recognition systems, providing a rigorous testbed for our framework.<br>We also appreciate the reviewer's recommendation of Khan et al.'s [17] work on bilateral feature fusion for handling diverse environmental conditions, which offers relevant insights for improving robustness across varying datasets.<br>We plan to extend our evaluation to UCF-101, HMDB-51, and ActivityNet in future work to comprehensively validate TinyAct's cross-dataset generalizability under diverse environmental conditions and action categories.
5	Comment 5: The 16-frame video clip processing with 1024-dimensional latent features shows	We appreciate the reviewer raising this point. We agree that our manuscript does not sufficiently analyze how frame

	promise for computational efficiency, but the paper does not provide sufficient analysis of how frame sampling strategies affect recognition accuracy for different action types with varying temporal dynamics. ***Please take assistance from the study titled "optimal features driven hybrid attention network for effective video summarization for developing adaptive frame sampling and temporal attention mechanisms.***	sampling strategies affect recognition accuracy across action types with different temporal characteristics.<br>Our current approach uses fixed 16-frame uniform sampling, which we selected as a practical trade-off between temporal coverage and the computational budget of edge devices. While this achieves 57% accuracy on Kinetics-400, we recognize that a single fixed sampling rate is suboptimal. Fast actions like clapping likely need denser frames than slow actions like stretching.<br>The reviewer's suggested reference [30] on hybrid attention for video summarization is relevant here, as it shows how temporal attention can adaptively weight frames based on their informativeness. Adapting this idea to action recognition, where the model learns which frames matter most for each action type, is a promising direction.<br>That said, properly implementing and benchmarking adaptive sampling across 400 action categories would require substantial additional experimentation beyond the scope of this revision. We have noted this direction in our Future Work section (Page 20, Lines 770–771), where we discuss extending the encoder to handle varying temporal dynamics through hierarchical modeling.
6	<u>Comment 6:</u> The comparison with SVM (55.00%) and XGBoost (54.00%) baselines provides context, but the paper lacks comparison with other state-of-the-art lightweight action recognition methods specifically designed for edge deployment, limiting the assessment of relative performance. Please take assistance from the study titled ***"efficient deepfake detection via layer-frozen assisted dual attention network for consumer imaging devices"*** for comparative analysis with other efficient detection frameworks on edge devices.	We thank the reviewer for this important observation. We agree that comparing TinyAct only against SVM and XGBoost baselines provides insufficient context for assessing its relative performance among lightweight action recognition methods. Following the reviewer's suggestion and drawing on the comparative framework established by Usman et al. [16] for evaluating efficient architectures on consumer edge devices, we have made the following additions:<br><u>CHANGES IN MANUSCRIPT:</u><br>1. REFERENCE [16] already included (Page 22)<br>Also added X3D [32] and SlowFast [33] as new references (Page 23).<br>2. ADDED NEW COMPARISON TABLE AND DISCUSSION<br>Location: Page 18, Section "Comparison with Lightweight State-of-the-Art Methods", Lines 674–696<br>Added Table 3 (Page 18) comparing TinyAct against five representative lightweight SOTA methods on Kinetics-400: MoViNet-A0 [21], TSM-ResNet18 [22], R(2+1)D-18 [23], X3D-XS

		[32], and SlowFast-R18 [33], across Top-1 Accuracy, Parameters (M), GFLOPs, Inference Time (ms), and Memory (MB).<br>Added a discussion paragraph (Page 18–19, Lines 675–696) contextualizing the accuracy gap (57.0% vs. 69.5–73.9%). Two key caveats are noted: first, SOTA methods use end-to-end inference on a single device while TinyAct splits feature extraction (edge) from classification (cloud), representing different deployment paradigms. Second, SOTA inference times are from server GPUs (V100/A100) while TinyAct reports encoder-only latency on Jetson Xavier NX. TinyAct's encoder (6.8M params, 2.1 GFLOPs) is comparable in complexity to lightweight models, but its split architecture enables multi-stream processing on edge hardware where models requiring 33.7M parameters and 128MB memory would not fit. TinyAct thus addresses a deployment scenario complementary to E2E methods: bandwidth-constrained, multi-camera AIoT environments.
7	<u>Comment 7:</u> The modular architecture design enabling flexible deployment across diverse hardware configurations is a significant strength. However, the paper does not provide concrete examples of deployment on specific edge devices or performance metrics on actual hardware platforms. Please take assistance from the study titled ***"lightweight transformer-driven multi-scale trapezoidal attention network for saliency detection"*** for practical deployment strategies on resource-constrained devices.	We fully acknowledge this limitation. The current manuscript provides computational profiling and theoretical deployment projections but does not include empirical measurements on physical edge hardware. We agree this is an important gap.<br>To address this transparently, we have added a new section "System Scalability and Deployment Feasibility" (Page 19, Lines 698–720) where we lay out detailed projections for three hardware tiers: Jetson Xavier NX handling 8–12 streams, Jetson Nano at 2–4 streams, and Raspberry Pi 4 for single-stream processing. We also discuss the encoder's memory footprint (27MB in FP32, 7MB in INT8) and potential optimizations such as INT8 quantization and TensorRT acceleration. Importantly, we state clearly in Lines 718–720 that empirical deployment validation, including latency profiling, thermal analysis, and stress testing, has not been conducted and remains essential for production readiness.<br>In our Future Work (Page 20, Lines 766–767), we have made hardware validation the first priority item, following deployment methodologies from [18] as the reviewer suggested.<br>We want to be upfront about why hardware experiments are not included in this revision. This study is primarily an algorithmic contribution covering lightweight architecture design, knowledge distillation for temporal modeling, and the cloud-edge split paradigm. Running proper hardware benchmarks

		across multiple device families with power measurement and thermal profiling requires infrastructure and testing time that falls outside the scope of this work. We are committed to pursuing this in a follow-up study.<br>We believe this treatment is honest about what we have and have not validated, while making clear that hardware deployment is our top priority going forward.
	Comment 8 : The integration of privacy-preserving data management through local edge processing is valuable for surveillance applications, but the paper does not address potential privacy concerns when compressed features are transmitted to the cloud for classification. Please take assistance from the study titled "bilateral feature fusion with hexagonal attention for robust saliency detection under uncertain environments" for developing robust feature representations that maintain privacy while preserving essential information.	We sincerely thank the reviewer for raising this important concern. We agree that our original manuscript emphasized the privacy benefits of edge processing but did not adequately discuss the risks of transmitting compressed latent features to the cloud. Following the reviewer's suggestion, we studied Khan et al. [17] on bilateral feature fusion and incorporated relevant insights throughout the manuscript.<br>CHANGES IN MANUSCRIPT:<br>1. REFERENCE [17] already included (Page 22)<br>Khan H, Usman MT, Koo J. Bilateral feature fusion with hexagonal attention for robust saliency detection under uncertain environments. Information Fusion. 2025;121:103165.<br>2. NEW SECTION: "Privacy Considerations in Cloud-Edge Architecture" (Page 19, Lines 721–735)<br>We added a dedicated section analyzing both the privacy advantages and remaining risks of our architecture. On the positive side, transmitting 1024-dimensional latent vectors instead of raw video provides baseline privacy protection through 99.9% dimensionality reduction (from 1,048,576 pixel values to 1,024 features), combined with multiple nonlinear transformations that make it difficult to recover original frames. Knowledge distillation also guides the encoder toward action-discriminative patterns rather than identity-revealing appearance details.<br>On the other hand, we honestly acknowledge that we do not claim formal privacy guarantees. The latent vectors still contain structured information that could be exploited through feature inversion or membership inference attacks. We describe this as a pragmatic improvement over raw video, not a privacy-certified solution.<br>3. ENHANCED RELATED WORK (Page 4, Lines 98–105)<br>Added a paragraph in the Lightweight Neural Networks subsection discussing how Khan et al. [17] demonstrated that selective attention mechanisms can extract robust, task-

		relevant features while maintaining resilience to environmental uncertainties. We extend this principle to privacy preservation: by focusing on action-discriminative patterns through knowledge distillation rather than preserving all visual details, compact feature representations can inherently emphasize task-relevant information over potentially sensitive identity-revealing features.<br>4. ENHANCED FUTURE WORK (Page 20, Lines 772–774)<br>Added formal privacy mechanisms as a future direction, including differential privacy, adversarial training against reconstruction attacks, and federated distillation that eliminates cloud transmission entirely.<br>5. UPDATED FIGURE 1 CAPTION (Page 7)<br>Revised the caption to include a note that facial regions in illustrative video frames have been obscured, and added text in the architecture summary (Page 7–8, Lines 258–268) acknowledging that compressed feature transmission (4KB per clip, 78–99× smaller than raw video) provides both computational efficiency and baseline privacy protection through feature abstraction.
	Comment 9 : The real-time monitoring interface and dashboard visualization components enhance practical applicability, but the paper lacks discussion of system scalability when handling multiple concurrent video streams or network latency effects on performance. Please take assistance from the study titled ***"efficient deepfake detection via layer-frozen assisted dual attention network for consumer imaging devices"*** for handling multiple stream processing efficiently on consumer devices.	We thank the reviewer for highlighting this critical gap in our practical deployment discussion. We have addressed this concern by adding substantial analysis of system scalability and network performance, incorporating insights from the suggested reference [16].<br>CHANGES IN MANUSCRIPT:<br>1. ADDED NEW SECTION: "System Scalability and Deployment Feasibility"<br>Location: After Privacy Considerations, before Conclusion (Page 19, Lines 698–720)<br>This section provides detailed scalability analysis based on computational profiling of TinyAct's encoder (6.8M parameters, 2.1 GFLOPs per clip). Key findings include: batch inference over K concurrent streams as tensors [K, 16, 3, 224×224] yields approximately 3.2× throughput improvement on Jetson Xavier NX (21 TOPS), with practical capacity at 5–7 streams at 10 FPS. We break down system latency into three components: feature transmission (4KB per clip, 78–99× smaller than raw video), cloud classification (5–15ms), and network round-trip (1–200ms). We also project performance across three hardware tiers: high-end (Jetson Xavier NX, 8–12 streams), mid-range

		(Jetson Nano, 2–4 streams), and low-power (Raspberry Pi 4, single-stream at 2–4 FPS). Optimization paths via INT8 quantization, TensorRT acceleration, and channel pruning are discussed. We explicitly acknowledge that empirical hardware validation has not been conducted and remains a primary future direction.<br>2. EXPANDED AIoT ARCHITECTURE SECTION<br>Location: Page 7–8, Lines 258–268<br>Added a summary paragraph detailing multi-stream processing capability (5–7 concurrent cameras via GPU-parallelized batch inference), the round-robin collection strategy (every 10 seconds per camera), compressed feature transmission (1024-dimensional vectors = 4KB per clip), and horizontal scaling through multiple edge devices with a local aggregation server for deployments exceeding single-device capacity.<br>3. REFERENCE [16] already included (Page 22)<br>Usman MT, Khan H, Singh SK, Lee MY, Koo J. Efficient deepfake detection via layer-frozen assisted dual attention network for consumer imaging devices. IEEE Transactions on Consumer Electronics. 2024;71(1):281–291.<br>4. ENHANCED FUTURE WORK (Page 20, Lines 766–767)<br>Hardware validation is prioritized as the first future work item, including latency profiling on physical edge platforms (Jetson family, Raspberry Pi) with stress testing.
	<u>Comment 10:</u> The overall contribution advances the field by demonstrating that effective human action recognition can be achieved without computationally intensive deep networks on edge devices. However, the evaluation needs strengthening through more comprehensive hardware testing and broader dataset validation. Please take assistance from the study titled “lightweight transformer-driven multi-scale trapezoidal attention network for saliency detection” for comprehensive evaluation methodologies on lightweight architectures.	We appreciate the reviewer's recognition of TinyAct's contribution and fully agree that comprehensive hardware testing and broader dataset validation would strengthen the evaluation. We have acknowledged these limitations in the revised manuscript.<br>The "System Scalability and Deployment Feasibility" section (Page 19, Lines 698–720) explicitly states that our scalability analysis is based on computational profiling, and that empirical validation on physical hardware remains as future work. The evaluation currently focuses on Kinetics-400 as our primary benchmark.<br>Following evaluation methodologies from [18] as suggested, we have prioritized these aspects in Future Work (Page 20, Lines 766–774) with detailed plans for hardware profiling on diverse platforms (Jetson Xavier/Nano, Raspberry Pi, Coral), optimization strategies (INT8 quantization, TensorRT acceleration), and multi-dataset validation.

		We view this study as establishing the algorithmic foundation: lightweight autoencoder-based feature extraction, knowledge distillation for temporal modeling, and the cloud-edge split paradigm. The comprehensive hardware and dataset evaluation suggested by the reviewer will be pursued in our subsequent research to advance this work toward production-ready deployment.<br>Thank you for this valuable guidance on strengthening the evaluation framework.

Part C: Technical Questions

Question 1: How does the knowledge distillation process specifically handle temporal dependencies during the teacher-to-student knowledge transfer, and what mechanisms ensure that critical spatiotemporal features are preserved in the compressed representation? Please take assistance from the study titled "optimal features driven hybrid attention network for effective video summarization" for temporal dependency modeling.

Response:

We thank the reviewer for this important question. Our manuscript addresses temporal dependency handling through two dedicated subsections added in the revised version.

Handling Temporal Complexity in Knowledge Distillation (Page 13–14, Lines 476–533)

Our framework preserves temporal dependencies through four complementary mechanisms:

First, temporal feature alignment. The ILA-ViT-B/16 teacher captures temporal dependencies through its Implicit Spatio-Temporal attention (IST) blocks, which align semantically relevant regions across adjacent frames. The student learns to replicate this temporal understanding by minimizing the distillation loss between softened logits, where the temperature scaling factor (T=4) exposes the teacher's nuanced temporal reasoning across the 16-frame sequence.

Second, progressive temporal learning. The 3D convolutional structure in our VideoAutoEncoder3D naturally preserves temporal ordering. Each Conv3D layer with kernel size (3,3,3) processes both spatial (x,y) and temporal (t) dimensions simultaneously, enabling hierarchical temporal representations where early layers capture low-level motion patterns and deeper layers integrate these into high-level action semantics.

Third, hybrid feature refinement. The autoencoder encoder captures raw spatiotemporal patterns through 3D convolutions, yielding a 1024-dimensional latent vector that encodes compressed temporal information. The residual MLP classifier then learns to weight and refine these patterns based on the teacher's soft targets, maintaining long-range temporal dependencies through residual connections in each MLPResBlock.

Fourth, reconstruction as temporal regularization. The reconstruction loss forces the decoder to recover the original 16-frame sequence from the latent representation, ensuring that the encoder maintains temporal structure even under the influence of distillation supervision. The balance between classification accuracy (via hard and distillation loss) and temporal fidelity (via reconstruction loss) is controlled by loss weights (α=0.5, β=0.5, δ=0.5).

Temporal Knowledge Transfer Strategy (Page 15–16, Lines 568–594)

We adopt a staged training approach. During the warm-up phase (first 20 epochs), the student learns basic spatiotemporal features through reconstruction and ground-truth supervision only, with β gradually increasing from 0 to 0.5. This allows the 3D convolutional encoder to establish stable temporal representations before attempting to mimic the teacher's complex temporal modeling. In the full distillation phase, teacher-student alignment refines temporal understanding at T=4, which we found empirically to provide the best trade-off between preserving nuanced temporal distinctions and maintaining training stability.

A key finding supporting this design: non-pretrained students (15.11% accuracy) outperform frozen pretrained encoders (5.82%), confirming that encoder-classifier co-adaptation is critical for capturing task-specific temporal dependencies that fixed spatial features from unsupervised reconstruction cannot provide (Table 2, Page 18).

The reviewer's suggested reference [30] on hybrid attention for video summarization informed our approach to channel-wise and spatial-wise feature refinement for temporal patterns, as discussed in Lines 507–520.

Question 2: What is the impact of network latency and bandwidth limitations on the system performance when transmitting compressed features from edge devices to the cloud, and how does the system handle situations where cloud connectivity is intermittent or unavailable? Please take assistance from the study titled “bilateral feature fusion with hexagonal attention for robust saliency detection under uncertain environments” for developing robust communication strategies under uncertain network conditions.

Response:

We thank the reviewer for this practical question regarding network performance and connectivity resilience.

Network Latency and Bandwidth Analysis

Our System Scalability section (Page 19, Lines 698–720) breaks down the end-to-end latency into three components: feature transmission from edge to cloud, cloud-side classification, and network round-trip. The critical design advantage is that TinyAct transmits only a 1024-dimensional latent vector (4KB per clip) rather than raw video frames, achieving 78–99× bandwidth reduction. This means even on moderate-bandwidth connections (1–10 Mbps per stream), feature transmission adds negligible latency compared to raw video streaming.

Cloud classification takes approximately 5–15ms, and network round-trip varies from 1–200ms depending on deployment conditions. For small-scale deployments (1–5 cameras), total system latency is estimated at 50–100ms, which is within acceptable bounds for surveillance and monitoring applications where per-clip rather than per-frame response is sufficient.

The compact 4KB transmission also provides natural resilience to bandwidth fluctuations. Where raw video streaming would degrade or fail under constrained bandwidth, a 4KB packet can be transmitted reliably even on low-quality connections. This is a practical advantage that follows from our architecture design rather than an explicit optimization.

Intermittent or Unavailable Cloud Connectivity

We acknowledge that the current manuscript does not address offline or intermittent connectivity scenarios. Our architecture assumes a persistent edge-to-cloud connection for classification. When connectivity is lost, the edge device can continue encoding and storing latent vectors locally (each clip requiring only 4KB), but classification would be delayed until connectivity resumes.

A more robust solution would involve deploying a lightweight fallback classifier directly on the edge device for critical applications. Given that our latent vectors already work with simple classifiers like Random Forest (57% accuracy with base autoencoder features, Table 1), running such a classifier on-device during connectivity loss is technically feasible. However, we have not implemented or evaluated this fallback mechanism.

The reviewer's suggested reference [17] on robust feature fusion under uncertain environments is relevant here. Khan et al.'s approach to maintaining performance under environmental uncertainties offers a useful framework for thinking about communication uncertainty as well. Developing robust transmission strategies, including local buffering, priority-based feature queuing, and edge-side fallback classification, is an important future direction that we have noted in our revised manuscript.

We recognize this is a gap in the current work and plan to explore connectivity-resilient deployment strategies in subsequent research.

Part D: Response to Reviewer #2

Comment		Response
1	Citation [1] in the first line of introduction makes no sense.	We thank the reviewer for identifying this error. In the original submission, citation [1] in the first line of the Introduction was incorrectly assigned and did not correspond to a relevant action recognition reference.<br>We have corrected this by replacing it with:<br>Zhang K, Wang W, Zhang L, Qiao Y. Deep learning for video-based human action recognition: recent advances, datasets, and challenges. Multimedia Tools and Applications. 2017;76(21):23383–23412.<br>This review paper provides comprehensive coverage of deep learning approaches for video-based action recognition, making it a more appropriate reference for introducing the field in the opening sentence (Page 2, Line 2).
2	No need to write how authors validate their model in the introduction section it should be part of results section.	We thank the reviewer for this structural suggestion. In the revised manuscript, we have simplified the validation mention in the Introduction (Page 2, Lines 27–28) to state only that we evaluate our approach on the Kinetics-400 dataset, without reporting specific accuracy figures or detailed comparative results. All validation details, including accuracy metrics across classifiers (Table 1), knowledge distillation results (Table 2), and comparison with state-of-the-art methods (Table 3), are presented in the Results section (Pages 17–18) where they belong.
3	Author must write about manuscript breakdown in last paragraph of introduction like section 2 represents literature review section 3 represents methodology etc.	We thank the reviewer for this suggestion. We have added a manuscript organization paragraph at the end of the Introduction section:<br>" The remainder of this paper is organized as follows. Section 2 reviews related work on lightweight architectures, real-time action recognition, and knowledge distillation. Section 3 presents the TinyAct methodology, including the AIoT architecture, autoencoder design, and knowledge distillation framework. Section 4 describes our experimental setup and presents results on the Kinetics-400 dataset. Section 5 discusses system scalability, privacy considerations, and the knowledge distillation performance gap. Finally, Section 6 concludes the paper and outlines future research directions."
4	Author must include more details in Transformer-Based Architectures for Action Recognition, Self-Supervised Learning for Action	We thank the reviewer for these editorial suggestions. We have made the following changes:<br>1. Subsection headings in Related Work (Page 4–5)

	Recognition otherwise dont write them as heading and convert all data in simple paragraph style.<br>Unbold Knowledge Distillation (KD) on line 133.	We chose to retain the subsection headings ("Transformer-Based Architectures for Action Recognition", "Self-Supervised Learning for Action Recognition", "Lightweight Architectures for Efficient Temporal Modeling") and expanded the content under each to provide sufficient detail. Each subsection now includes discussion of representative methods, their strengths, limitations, and how TinyAct differs. We believe this organization improves readability given that the Related Work covers three distinct research streams under section "(2) Real-Time Action Recognition in Video Analysis" (Page 4, Line 121).<br>2. Unbolded KD heading (Page 5, Line 166)<br>Changed the formatting of "(3) Knowledge Distillation for Model Compression" from bold section heading to regular paragraph-style text, consistent with the reviewer's preference.
5	Author should merge Training Procedure and Hyperparameter Settings in Training Configuration and Implementation Details because both looks same otherwise specify the difference between then and need to write them in two different places.	We thank the reviewer for this organizational suggestion.<br>We have merged the sections as recommended:<br>Changed heading from:<br>"Training Procedure and Hyperparameter Settings"<br>To:<br>"Training Configuration and Implementation Details"<br>The merged section now provides a unified, streamlined presentation of all training-related information without redundancy, improving the manuscript's readability and logical flow.
6	Authors mentioned results of their own experimentations and did not perform SOTA comparison in this study which needs to include.	We thank the reviewer for this comment. We agree that SOTA comparison is essential for positioning TinyAct within the broader landscape of action recognition methods.<br>In the revised manuscript, we have added a new subsection "Comparison with Lightweight State-of-the-Art Methods" (Page 18, Lines 674–696) with Table 3 comparing TinyAct against five representative lightweight methods on Kinetics-400: MoViNet-A0 [21], TSM-ResNet18 [22], R(2+1)D-18 [23], X3D-XS [32], and SlowFast-R18 [33]. The comparison covers Top-1 Accuracy, Parameters (M), GFLOPs, Inference Time (ms), and Memory Usage (MB).<br>The accompanying discussion (Pages 18–19) contextualizes the accuracy gap (57.0% vs. 69.5–73.9%) by noting two important caveats. First, SOTA methods perform end-to-end inference on a single device, while TinyAct splits feature extraction (edge) from classification (cloud), so these represent fundamentally different deployment paradigms. Second, SOTA inference times are

		measured on server-class GPUs (V100/A100), whereas TinyAct reports encoder-only latency on a Jetson Xavier NX, making direct latency comparison inappropriate.<br>In terms of model complexity, TinyAct's encoder (6.8M parameters, 2.1 GFLOPs) falls between the lightest models like MoViNet-A0 (3.1M) and heavier architectures like SlowFast-R18 (33.7M). TinyAct's value lies not in matching SOTA accuracy, but in enabling multi-stream edge deployment on bandwidth-constrained AIoT environments where models requiring 33.7M parameters and 128MB memory would not fit.
